# Geodesign to advance boundary work in urban planning: A study in Stockholm focused on nature-based solutions

**DOI:** 10.1007/s13280-024-02083-8

**Published:** 2024-10-19

**Authors:** Blal Adem Esmail, Cyrus Carl Anderson, Sigvard Bast, Chiara Cortinovis, Lina Suleiman, Jarumi Kato-Huerta, Johan Högström, Berit Balfors, Gustavo Arciniegas, Davide Geneletti, Ulla Mörtberg, Christian Albert

**Affiliations:** 1 Institute of Geography, Ruhr University Bochum, Universitätsstr. 150, 44805 Bochum, Germany; 2 Institute of Environmental Planning, Leibniz Universität Hanover, Herrenhäuser Str. 2, 30419 Hanover, Germany; 3 Department of Sustainable Development, Environmental Science and Engineering, KTH Royal Institute of Technology, Teknikringen 10B, SE-10044 Stockholm, Sweden; 4 Department of Civil, Environmental and Mechanical Engineering, University of Trento, 38123 Trento, Italy; 5 Department of Urban Planning and Environment, KTH Royal Institute of Technology, 10044 Stockholm, Sweden; 6 Geo-Col GIS and Collaborative Planning, 2612 BB Delft, The Netherlands; 7 GLOMOS - Centre for Global Mountain Safeguard Research & Institute for Alpine Environment, Eurac Research, Bolzano, Italy

**Keywords:** Boundary management, Impact assessment, Knowledge co-creation, Land use change, Scenario planning, Suitability analysis

## Abstract

Geodesign supports collaborative urban planning by managing ‘boundaries’ between diverse knowledge holders. However, there is a paucity of empirical evidence of its contribution to ‘boundary work’. This paper aims to evaluate how a geodesign process facilitates knowledge co-production through boundary work and to assess the scientific credibility, political saliency, and procedural legitimacy of its outputs in urban planning. We propose a replicable geodesign framework to assess boundary work, and test it in a case study on urban transformations with nature-based solutions in the Skarpnäck district of Stockholm, Sweden. Findings indicate that all geodesign steps facilitated communication by promoting collective reasoning. Participants acknowledged contributions to knowledge co-production and decision-making by mediating between different perspectives. However, data quality and modeling simplicity were identified as critical factors affecting the outputs’ perceived credibility. Future applications should include co-designing the geodesign process, improving capacity and skills, and facilitating more integrated planning.

**Supplementary Information:**

The online version contains supplementary material available at 10.1007/s13280-024-02083-8.

## Introduction

Urban sustainability issues are inherently complex and challenging, often described as *wicked problems* (Brown et al. 2010; Orland 2016) due to intricate interlinkages between urban transformations, human well-being, biodiversity and climate change (Seto et al. 2012; McDonald et al. 2020). In addition, addressing these issues requires engagement with diverse and multifaceted processes that span across sectors and scales (Geels 2011; Collier et al. 2013; Suleiman and Khakee 2017). Urban planning is an example of such processes, involving collaboration and co-creation among stakeholders (e.g., Albert et al. 2014; Geneletti et al. 2020). Urban planners, as facilitators and knowledge brokers, can play a pivotal role in addressing sustainability issues by leveraging the full potential of urban areas. However, two main interconnected challenges for planners are centered on integrating diverse data and knowledge, and facilitating collaboration across governance levels, competencies, and sectors (Frantzeskaki et al. 2020; Sarabi et al. 2022; Kauark-Fontes et al. 2023).

Integrating diverse data and knowledge into planning processes at local and regional levels presents a complex challenge. Planning for multiple demands, such as new housing development and green spaces, requires additional data and knowledge that must be integrated into already extensive existing datasets (Cortinovis and Geneletti 2020). The assessment of the multidimensional impacts of proposed interventions is also necessary to inform the iterative planning and design process itself. This complexity highlights the need for Planning Support Systems (PSS), which are a subset of computer-based geo-information tools aiding planners in managing and exploring their activities (Geertman and Stillwell 2004). PSS components include datasets, algorithms, display facilities, theoretical constructs, knowledge, and modeling capabilities. They aid the planning process by communicating information and generating solutions (Geertman and Stillwell 2020).

Recently, the notion of PSS has become intertwined with geodesign, an emerging planning paradigm advocating for the integration of digital tools with stakeholder design discussions (Campagna 2022). As a methodology approach, geodesign *‘tightly couples the creation of design proposals with impact simulations informed by geographic contexts, systems thinking and digital technology*’ (Steinitz 2012, p. 12). Several studies have shown geodesign potential to integrate diverse data and knowledge and help resolve conflicts over future space use (e.g., Campagna & Di Cesare 2016 in Italy; Huang & Zhou 2016 in China; Rivero et al. 2017; Pettit et al. 2019 in Australia; Gottwald et al. 2021a, b in Germany; Moura & Freitas 2021 in Brazil; Schröter et al. 2023 in Costa Rica), making it a promising methodology approach for collaborative spatial planning to address urban sustainability issues.

Facilitating stakeholder collaboration is crucial for effectively integrating, interpreting, and communicating input data and outcomes within the planning process. One significant challenge is the presence of ‘boundaries’ between stakeholders, their knowledge systems, different planning levels, and various sectors (Parker and Crona 2012; Adem Esmail et al. 2017). These boundaries embody the shared understandings and strategies various interest groups and professions employ to bridge their knowledge domains and positions (Bartel and Garud 2009). Addressing urban sustainability issues requires ‘boundary work’ to reconcile diverse perspectives and interests (Adem Esmail and Geneletti 2017). As articulated by Cash et al. (2003a), boundary work refers to the efforts made by organizations or individuals to navigate the interface between diverse stakeholders engaged in co-producing knowledge. Several studies have demonstrated the fundamental role of boundary work in facilitating collaborative knowledge co-generation for sustainable development (van Kerkhoff and Lebel 2006; Mollinga 2010; Crona and Parker 2012; Clark et al. 2016; Adem Esmail et al. 2017).

Recent research indicates that ‘boundaries’ can act as dynamic shared interfaces where stakeholders can collaboratively address planning and management questions in a geodesign setting. For instance, Gottwald et al. (2021a, b) found geodesign effective in supporting boundary work functions such as *translation*, *communication*, and *mediation* in river landscape planning in Germany. However, as complexity increases, concerns arise regarding the perceived *credibility* and *legitimacy* of the geodesign process. Similarly, Schröter et al., (2023) found geodesign valuable and supportive to local stakeholders in virtual participatory mapping in Costa Rica's Grande de Tárcoles River basin. Despite these findings, questions remain about their transferability to other socio-ecological contexts and, more specifically, how different steps of the geodesign process and associated tools affect boundary work functions and participants’ perceptions. Advancing the field requires a deeper understanding of geodesign benefits and practicality in enhancing planning processes, as well as an assessment of the willingness of professionals to utilize such a methodology approach and related tools (Hooper et al. 2021).

Against this background, our aim is to evaluate how a geodesign process facilitates knowledge co-production through boundary work and assess the scientific *credibility*, political *saliency*, and procedural *legitimacy* of its outputs in urban planning for sustainability issues. The research questions are: (i) *How do participants perceive different geodesign steps in terms of their ability to facilitate the co-production of knowledge through boundary work?* (ii) *How do participants evaluate the outputs of the geodesign process in terms of their scientific credibility, political saliency, and procedural legitimacy?* To this end, we develop an integrated framework of geodesign and boundary work and a related protocol for assessing boundary work functions and criteria. The integrated conceptual framework and its operationalization through the methods for eliciting participants’ feedback are a first attempt to systematically incorporate the assessment of boundary work as a replicable protocol for geodesign applications in urban contexts. Ultimately, we seek to explore geodesign as a transformative tool for integrating data and knowledge and facilitating effective stakeholder collaboration in planning to address pressing urban sustainability issues.

To address our research questions, we focus on the example of planning with nature-based solutions (NBS). NBS are increasingly central in urban policy and planning discussions due to their potential to redefine the relationship between people and nature, thereby driving urban sustainability transitions (Adams et al. 2023). NBS offer significant opportunities for collaborative design practices that benefit both people and nature (Raymond et al. 2017; Frantzeskaki 2019; Anderson and Renaud 2021; Babí Almenar et al. 2021; McPhearson et al. 2023). However, their multifunctionality necessitates the integration of diverse data and knowledge. Additionally, conflicts often arise between the implementation of NBS and the demands for urban densification and housing, making NBS a prime example of challenges that require collaboration and boundary-spanning approaches (e.g., Sarabi et al. 2022). Therefore, planning with NBS provides a valuable test case for investigating the role of geodesign as a methodological approach that can help planners address the twin challenges of integrating data and knowledge and facilitating collaboration.

The city of Stockholm, particularly the Skarpnäck district, was chosen as the research site due to its relevance in planning urban transformation amidst population growth, limited land resources, climate change, and biodiversity loss (e.g., Adem Esmail et al. 2022; Brokking et al. 2021). Skarpnäck exemplifies inter-scalar planning issues, is the subject of ongoing formal planning processes, and planning stakeholders were willing to jointly explore alternative development options. A key planning question for the district is how to provide new housing while addressing issue of biodiversity loss, climate change, and urban quality of life. It thus allowed us to develop and examine a geodesign process case study, involving fourteen planning stakeholders from Stockholm, to evaluate the effectiveness of the geodesign process in addressing the twin challenges of integrating data and knowledge and facilitating collaboration across governance levels, competencies, and sectors.

## Geodesign process and boundary work: A conceptual framework

In his most established Geodesign Framework, Steinitz posits that a comprehensive geodesign process should comprise three iterations along six fundamental stages, or 'models' (Steinitz 2012). The three iterations serve: to understand the scope of the study (i.e., the *scoping* process), to define the detailed procedures (i.e., *metaplanning*), and to execute the geodesign study (i.e., *implementation*). Embedded in a specific socio-ecological and institutional context (e.g., Dabović, 2020), the entire geodesign process is not linear but entails feedback loops or alternative routes centered on the *people of the place* whose participation is of paramount importance throughout the process. Steinitz’s framework is conceptual in nature and must be adapted to context-specific research and/or planning questions to actually guide the implementation of geodesign processes (Kuniholm 2020). The latter are typically implemented through scenario-based workshops that use GIS interfaces, which serve as tools for crafting plausible and coherent future visions, which help foster anticipatory knowledge (Iwaniec et al. 2020).

**Fig. 1:**
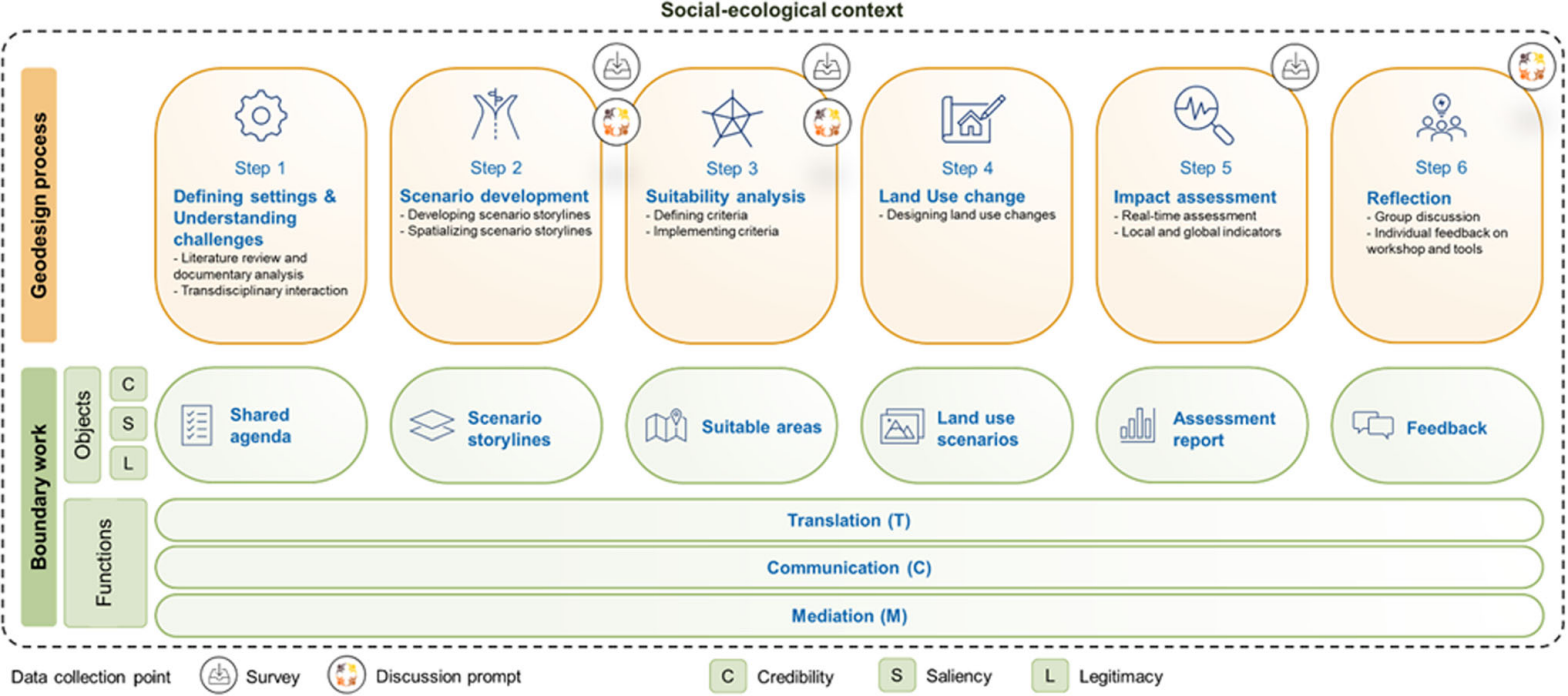
Conceptual framework of a 6-step geodesign process (upper panel) and its contribution to boundary work (lower panel). Adapted from Gottwald et al. 2021a, b. The last four steps of the geodesign process indicate those steps followed during the two-day workshop

Building on recent research efforts for operationalizing Geodesign for boundary work in planning with NBS (Albert et al. 2021; Gottwald et al. 2021a, b), we here propose a scenario-based geodesign process consisting of six core steps embedded in a given socio-ecological system (Fig. 1). *Step 1* involves co-defining the setting and understanding the challenges in the study area through transdisciplinary interactions and reviewing pertinent documents. *Step 2* entails jointly developing scenario storylines that embody distinct transformative goals, such as varying levels of NBS adoption. *Step 3* includes a suitability analysis that uses a user-friendly/customized GIS interface to identify areas most suitable for achieving urban development objectives, including new housing and NBS. *Steps 4 and 5* consist of the 'Land Use Change' and associated 'impact assessment' activities conducted by geodesign participants using the same GIS interface. Finally, *Step 6* involves participants in discussions, reflections, and evaluations. The practical implementation entails two types of workshops: a traditional workshop for creating scenario storylines tailored to the case study (*Step 2*), and a digital face-to-face workshop for implementing the final four steps of the geodesign process. This is aided by a customized GIS interface linked to a large touch-enabled screen to facilitate interaction.

Turning to boundary work, previous research has significantly advanced the operationalization of the concept. Key *attributes*, *effectiveness criteria*, and *functions* have been well-defined (Cash et al. 2003; Clark et al. 2016), and explored in empirical applications (e.g., Adem Esmail et al. 2017). Boundary work encompasses three key attributes. These involve meaningful stakeholder *participation* in setting agendas and producing knowledge, establishing governance mechanisms for *accountability*, and creating adaptable *boundary objects* that bridge different perspectives (Star and Griesemer 1989; Clark et al. 2016). The effectiveness of boundary work is evaluated using three criteria collectively known as CSL: *credibility* in handling scientific evidence, *salience* or relevance to the specific problem at hand, and *legitimacy* of the process, emphasizing fairness and respect for all involved (Cash et al. 2003; Mitchell et al. 2006). Thirdly, boundary work functions include translating complex concepts for mutual understanding (*translation*), communicating actively and inclusively (*communication*), and mediating to resolve conflicts among stakeholders (*mediation*), here collectively referred to as TCM (Cash et al. 2003; Clark et al. 2016). Although the concept of boundary work, and CSL in particular, has matured, its application remains fragmented and lacks systematic approaches. Additionally, best practices for data elicitation need to be integrated (Anderson et al., in prep.).

Examining our proposed geodesign process through the lens of boundary work, each of the six steps has specific requirements and practical implications (Fig. 1). Firstly, every step produces collaborative outputs or insights, such as a shared agenda (Step 1), relevant scenario storylines (Step 2), suitability maps (Step 3), and proposals for land use changes, along with assessments of their potential multidimensional impacts (Steps 4 and 5). These outputs can act as 'boundary objects' in the collaborative geodesign process and can be assessed based on participants' perceptions of their CSL. Additionally, each step of the geodesign process can contribute to the boundary work functions of TCM. Evaluating the extent of these contributions, as perceived by participants, is challenging yet crucial for understanding how geodesign supports boundary work in planning with NBS and planning more broadly. Finally, boundary work is a dynamic process influenced by the socio-ecological context in which it is embedded (Cash et al. 2003; Crona and Parker 2012; Parker and Crona 2012). It is crucial to understand the timing, feedback mechanisms, and trust that exist beyond the geodesign process since contextual factors shape the success and effectiveness of boundary work in the broader planning landscape (Adem Esmail et al. 2017; Gottwald et al. 2021a, b).

## Materials and methods

The research design consisted of four main components: first, defining the study area; second recruiting participants; third, setting up a customized GIS interface based on a case-specific application of the geodesign steps outlined in Fig. 1; and fourth, conducting a digital-face-to-face geodesign workshop and collecting feedback from participants on the different steps and tools used through surveys and focus group discussions.

### Defining the study area

**Fig. 2:**
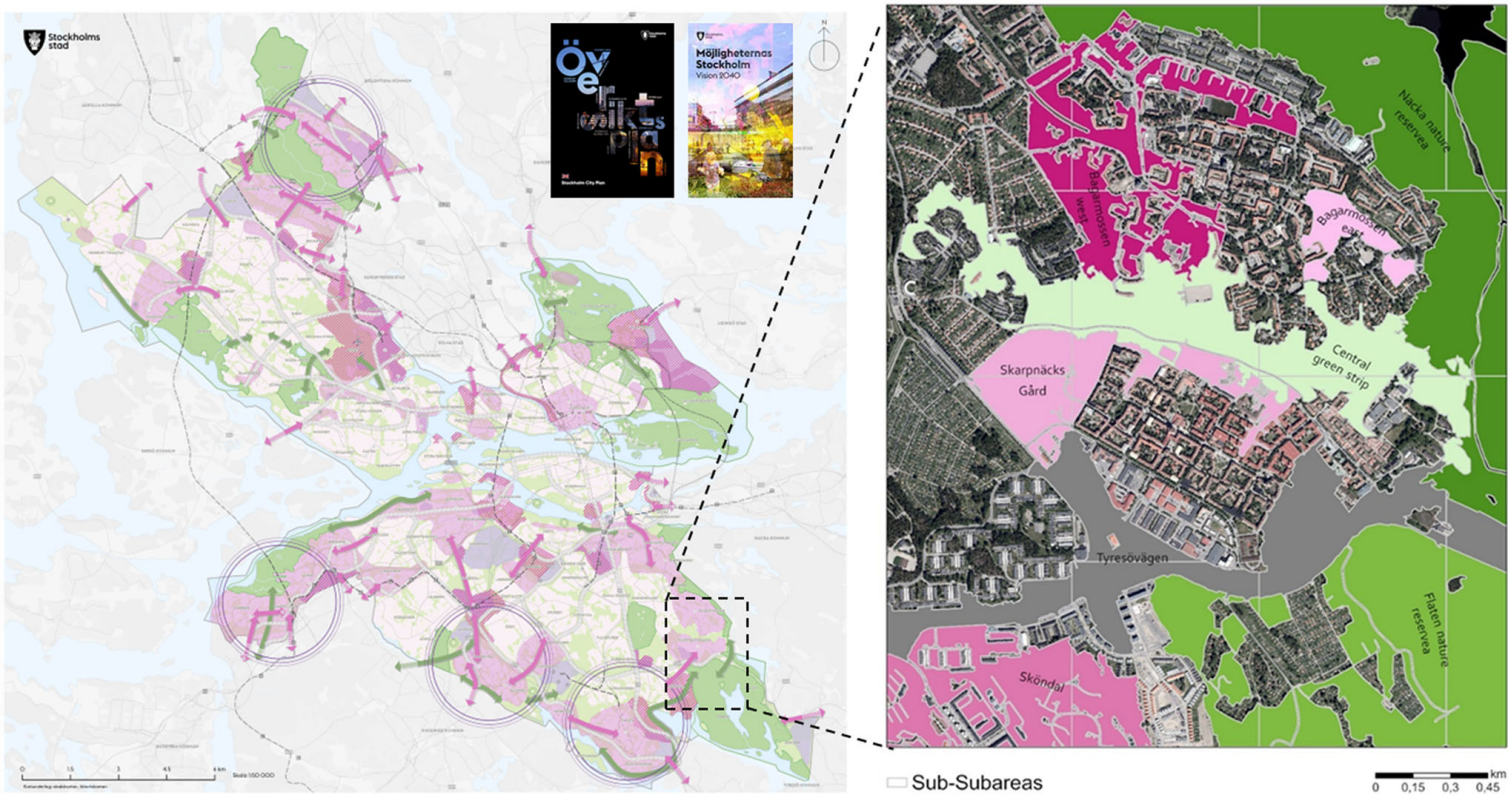
Study area defined in accordance with the 2018 Stockholm City Plan and its Vision 2040 (left), which identify areas of urban transformation and strategic social links (illustrated by pink hatches and arrows) and areas of nature conservation and ecological corridors (depicted by green hatches and arrows). The study area is subdivided into eight sub-areas within the Bagarmossen and Skarpnäcks Gård neighborhoods, identified as "opportunity spaces" for both housing and/or NBS development (right)

The case study focuses on a 750-hectare area in the Skarpnäck district, encompassing the Bagarmossen and Skarpnäcks Gård neighborhoods (Fig. 2). This selection was guided by the Stockholm City Plan and input from a planner working for the City of Stockholm. The study area presents opportunities to influence regional green infrastructure and foster important social connections at the local and city levels. Eight sub-areas within the study area were identified as 'opportunity spaces' for housing and/or NBS development. These include six areas with proposed plans and two adjacent nature reserves, avoiding locations with existing detailed plans. To facilitate communication and serve as a basis for local impact assessments, the sub-areas have been further subdivided into 27 sub-sub-areas based on a 750-m grid.

**Fig. 3:**
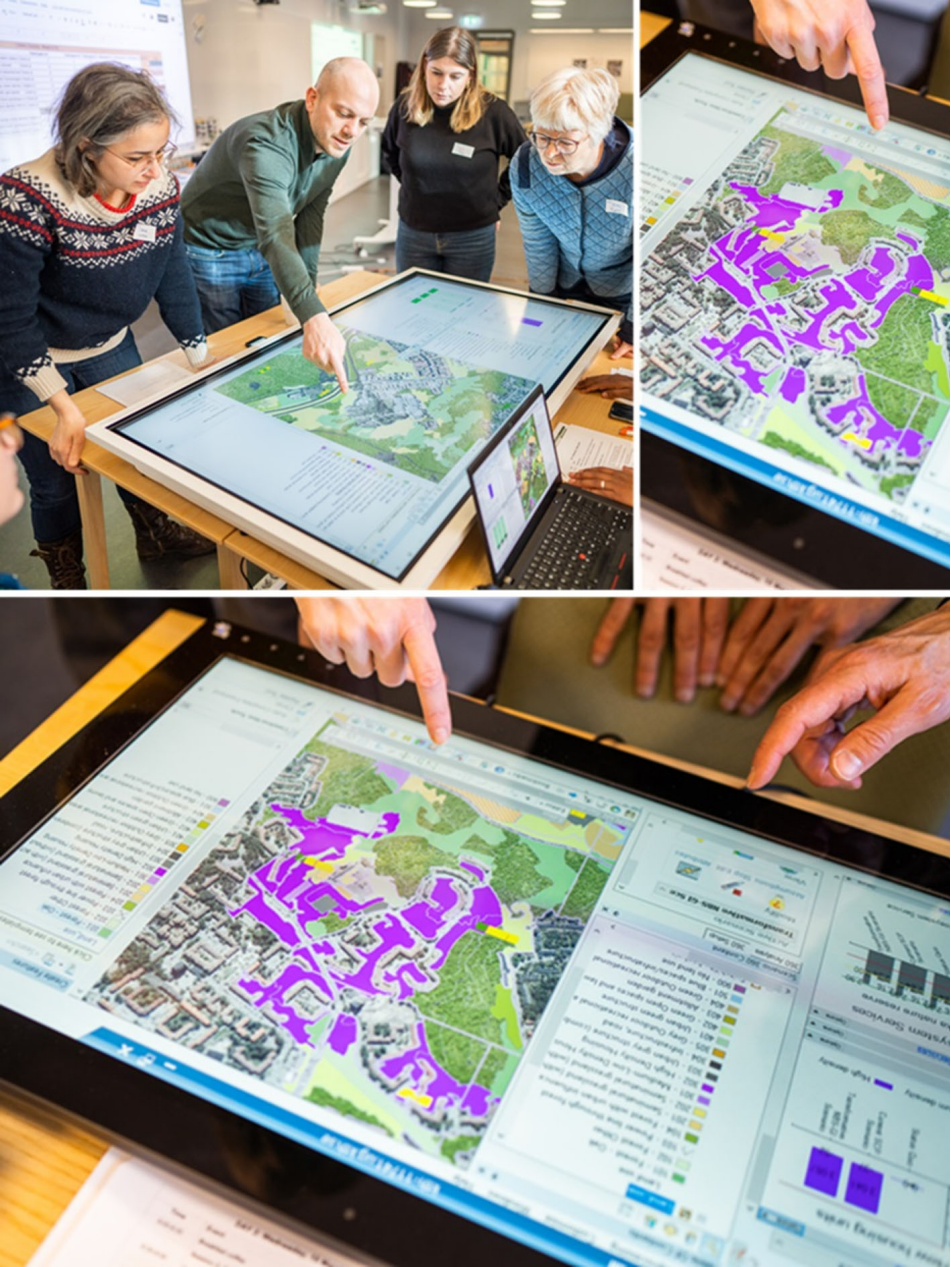
Geodesign workshop participants (Photo credit: Magnus Glans)

### Recruiting participants

The recruitment process was a collaborative effort between our research team and our contact at the City of Stockholm. The goal was to assemble a diverse group of participants representing various planning levels, from local to regional, and possessing expertise in sectors like climate change adaptation, biodiversity, and urban development. Fourteen experts participated in the two half-day Geodesign workshop, all well-versed in the political and operational aspects of the Swedish planning system and deeply aware of societal challenges facing Stockholm (Fig. 3).

These participants exhibited diverse boundaries across spatial scales (from municipal to national), competencies, and thematic interests related to social value, climate adaptation, and biodiversity protection. Most participants hold master's degrees in fields such as urban planning and design, environmental sciences, and architecture. Their professional roles span all planning/administrative levels (local, city, and regional) and include job titles such as regional climate adaptation coordinator, urban planner, and landscape architect. Their professional experience varies from 1 to 27 years, with some having worked at multiple levels (see Table S1 in SM).

**Table 1:** Criteria considered for the suitability analysis: Cr. 1–4 pertaining to the built-up environment and Cr. A–D to the natural environment. For each criterion, the arrows indicate the suitability direction: ↑ means the higher the value, the better; ↓ means the lower the value, the better

	Name	Criteria	Suitability direction	Data source
Cr. 1	Metro	Distance from two metro stations	↓	(Lantmäteriet 2022)
Cr. 2	Schools	Distance from schools	↓	(Lantmäteriet 2022)
Cr. 3	Industrial buildings	Distance from industrial buildings	↑	(Lantmäteriet 2022)
Cr. 4	Tyresövägen	Distance from Tyresövägen	↑	(Lantmäteriet 2022)
Cr. A	Tree Canopy	Tree canopy cover density (100 m)	↑	(Stockholm City 2022)
Cr. B	Oak habitat	Overlap with oak habitat	↓	(CAB 2019)
Cr. C	NB-recreation	Distance from sociotopes with high value for NB-recreation	↓	(Ståhle 2003)
Cr. D	Imperviousness	Impervious density (100 m)	↓	(EU Copernicus LMS and EEA 2018)

### Setting up a customized GIS interface for the Skarpnäck case study

**Fig. 4:**
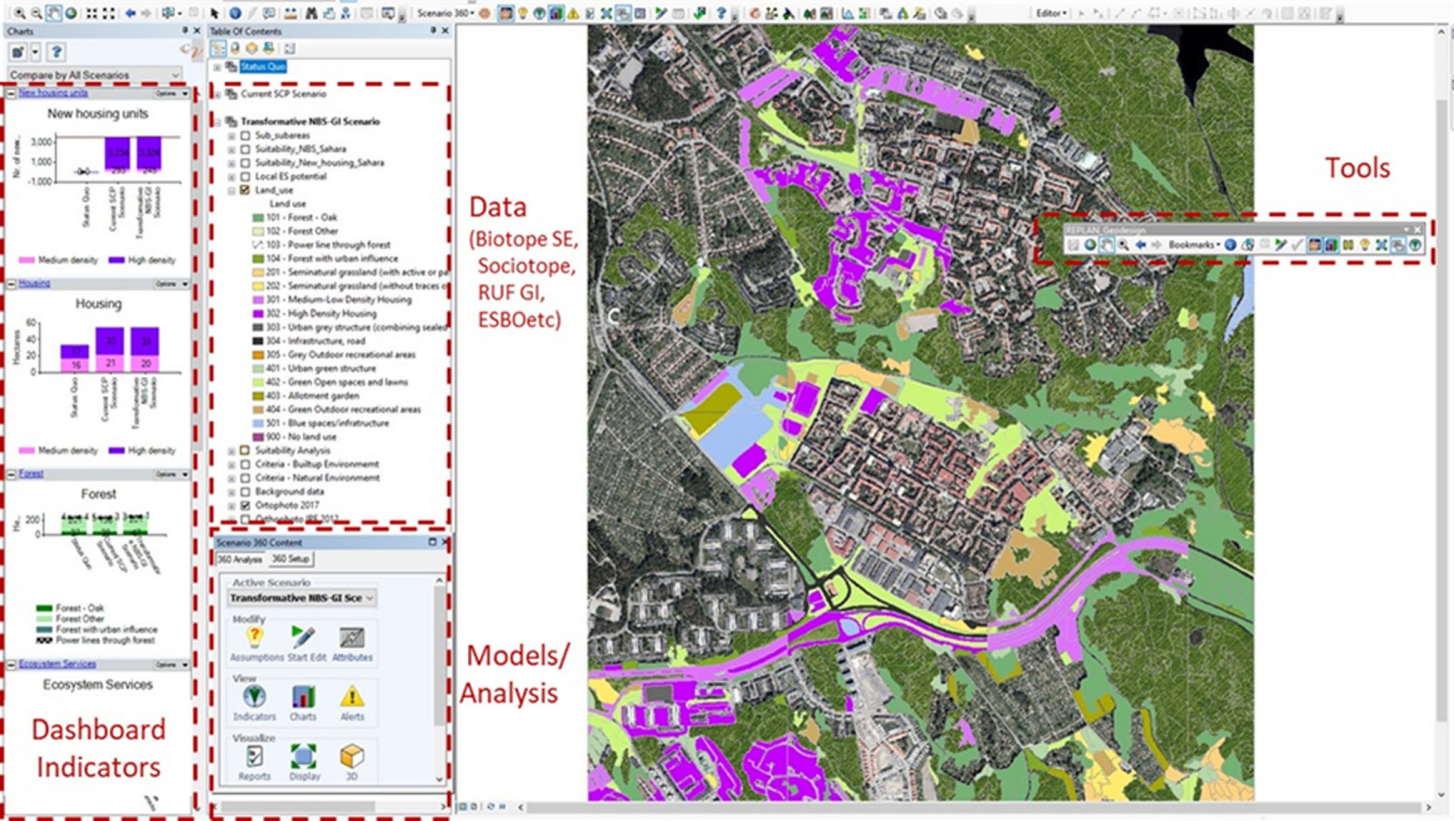
GIS Interface of the study area: data, tools, models, and indicator dashboards

A customized GIS interface was developed using ArcGIS 10.8.1 and the CommunityViz V2 extension (Fig. 4). This interface integrated spatial data, geographic calculation tools, assessment models, and indicator dashboards to support the geodesign process. It provided tools for conducting suitability analysis (*Step 3*), land use change (*Step 4*), and impact assessment (*Step 5*).

Information gathered during *Steps 1* and *2* guided the development and setup of the GIS interface. *Step 1* involved co-defining the setting and understanding challenges by reviewing the 2018 Stockholm City Plan and its Vision 2040, drafting a summary, and consulting key planning stakeholders. A primary challenge identified was accommodating a significant population increase in the Skarpnäck district while enhancing social and ecological connectivity. *Step 2*, *Scenario development*, entailed downscaling and adapting the official vision for Stockholm to the context of Skarpnäcks Gård and Bagarmossen, resulting in two scenarios: a *Current Stockholm City Plan Scenario* (SCP) and a *Transformative Change Scenario* (TC) emphasizing biodiversity and ES.

In *Step 3*—*Suitability Analysis*, participants focused on assessing suitability for new housing and NBS based on main challenges and opportunities identified in the Stockholm City Plan. The assessment employed eight criteria pertaining to the built-up environment (Cr. 1–4) and the natural environment (Cr. A-D), which had been developed by the research team and implemented within the GIS interface (Table 1). Participants could explore maps of these criteria and combine them by assigning weights from 0 to 10 to identify areas most suitable for new housing and NBS. Additionally, the GIS interface included background spatial information like biotope and sociotope maps, protection statuses, green infrastructure, settlements, and ES, aiding participants in interactive navigation in the GIS (see Table S2 in SM). A detailed description of the spatial multicriteria methods for the suitability analysis can be found in CommunityViz Tutorials—Use with CommunityViz 5.2.

**Table 2:** Structure of the implementation 2-half-days’ workshop. Workshop sessions and characteristics aligned with geodesign process (Q = questionnaire; FGD = Focus Group Discussion; TCM = Translation, Communication, Mediation; SCP = Stockholm City Plan; TC = Transformative Change; CSL = Credibility, Salience, Legitimacy)

Workshop session	Description	Geodesign step addressed	Questionnaire (Q) and FGD prompt (D)	Concepts assessed
Pre-workshop		Steps 1, 2	Q1. Pre-workshop survey	Background, TCM
Session 1 (20 min)	Introduction			TC
Session 2 (60 min)	Discussing scenarios	Step 2	FGD1. Discussion prompt	TC
Session 3 (80 min)	Suitability analysis task: housing and need for NBS	Step 3	Q2. Survey	TCM (baseline and post-task)
Session 4 (50 min)	Plenary discussion	Step 3	FGD2. Discussion prompt	CSL
Session 5 (20 min)	Recap			
Session 6 (70 min)	Land use change and impact assessment tasks (current STCP scenario)	Steps 4, 5		
Session 7 (90 min)	Land use change and impact assessment tasks (transformative change scenario)	Steps 4, 5	Q3. Survey	TCM
Session 8 (50 min)	Reflections	Steps 4, 5, All steps	FGD3. Discussion prompt	CSL, general

In *Step 4*—*Land Use Change* and *Step 5*—*Impact Assessment*, participants could modify existing land uses to meet the required number of new housing units and create new NBS, aligning with the two scenario storylines and outcomes of the previous suitability analysis. Land use data were sourced from the Biotope.se map (Skånes 2022) and adapted to address planning issues in the case study area. Participants could select polygons in the land use map and change their current land use. To facilitate negotiations among participants during the co-design process, the GIS interface provided real-time assessment of multidimensional impacts of proposed land use changes. An indicator dashboard (Fig. 4) presented key information related to planning goals, such as the number of additional high- and low-density housing units, ecosystem changes including hectares of oak and other forests, and selected ES. Indicators were displayed using a traffic light scale in both aggregated and disaggregated forms (see Figure S1 in the SM). The GIS interface facilitated comparative analysis and visualization of proposed scenarios against the status quo, enriching discussions, and deliberations in the geodesign process.

The analysis considered potential impacts on four selected ES indicators (local climate regulation, stormwater retention, habitat supporting biodiversity, nature-based recreation), chosen based on the relevant themes and societal challenges identified in *Step 1*. To assess the potential ES impacts, a look-up table method was applied, using values based on the best-available habitat data from Biotope.se (Skånes 2022). A Delphi approach was employed to elicit and reach consensus on the look-up table values (Mukherjee et al. 2015). The potential of each biotope within the Skarpnäck study area to provide selected ES was evaluated by the study team and the City of Stockholm contact person, using a scale of 0 (negligible) to 3 (high). An external expert reviewed the assessments, and a consensus was reached on the values. Finally, a potential value for each ES was assigned to every land use class, based on an area-weighted average by considering the biotopes within each land use class.

### Conducting the workshop and collecting participants’ feedback

The final four steps of the geodesign process were conducted via a digital face-to-face workshop, utilizing the customized GIS interface accessible on three touch tables. Spanning two half-days, the workshop comprised eight sessions, including introductions, working, and evaluation sessions (Table 2). These sessions were conducted either in plenary or in three smaller groups of 4 to 5 participants each to ensure active participation (A, B, and C), each provided with access to a touch table and an identical GIS interface. Geodesign sessions for each group were facilitated by at least two members of the research team, with a master's student taking notes.

To collect feedback from participants, we employed a combination of three questionnaires and three Focus Group Discussions (FGDs). Survey items and discussion prompts were informed by a systematic literature review on boundary work criteria of CS, as detailed in Anderson et al. (in preparation). Survey items related to TCM were generated based on the definitions of these concepts, considering various dimensions and their relevance within the geodesign workshop context. Data related to TCM were primarily collected through survey items using a 9-point Likert scale, administered during the workshop. For CSL assessment, discussion prompts supported by real-time Mentimeter polls with CSL-specific items, including some baseline related, were relied upon. A 5-point Likert scale was implemented in Mentimeter.com, pilot tested, improved, and applied.

Participants completed two brief paper-based questionnaires immediately after (and referring to) the geodesign tasks related to suitability analysis (*Step 3*) and land use change and impact assessment (*Steps 4* and *5*). Furthermore, a brief questionnaire was administered seven days prior to the workshop, which included sections for collecting background information and expectations. To enhance the differentiation among respondents within the small sample, Likert 1–9 ascending disagree-agree items were mainly utilized.

We conducted FGDs to evaluate how participants assessed the outputs of the geodesign process, considering them as ‘boundary objects’. The evaluation focused on the scientific credibility, political salience, and procedural legitimacy of the geodesign outputs. Two separate discussion activities took place at different stages of the geodesign process. The FGDs specifically addressed the perceived CSL of the outputs generated during the two main geodesign steps: suitability analysis (*Step 3*) and land use change and impact assessment (*Steps 4* and *5*). The discussions commenced with a live survey using a 1–5-point Likert scale through Mentimeter, which was used to stimulate discussion. This was followed by open-ended reflection questions. All FGDs were audio recorded, fully transcribed, and supplemented by the notetaking of three master students during the workshop. A copy of the questionnaires and FGD discussion prompts can be found in the Supplementary Material.

## Results

### Geodesign and knowledge co-production through TCM

**Fig. 5:**
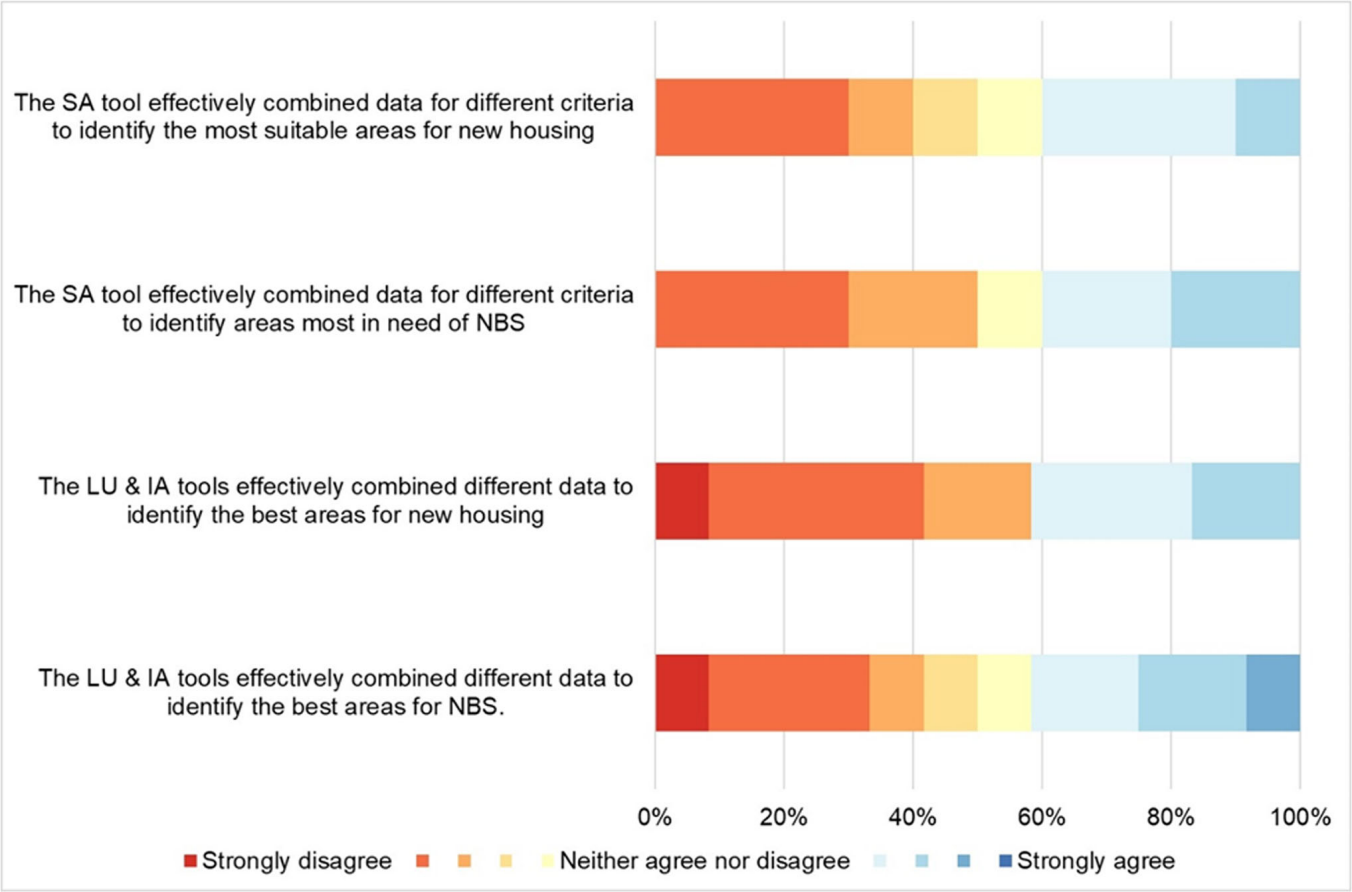
Perceived contribution of the ‘suitability analysis’ (SA) and ‘land use change and impact assessment’ (LU & IA) steps to facilitate knowledge co-production through translation

**Fig. 6:**
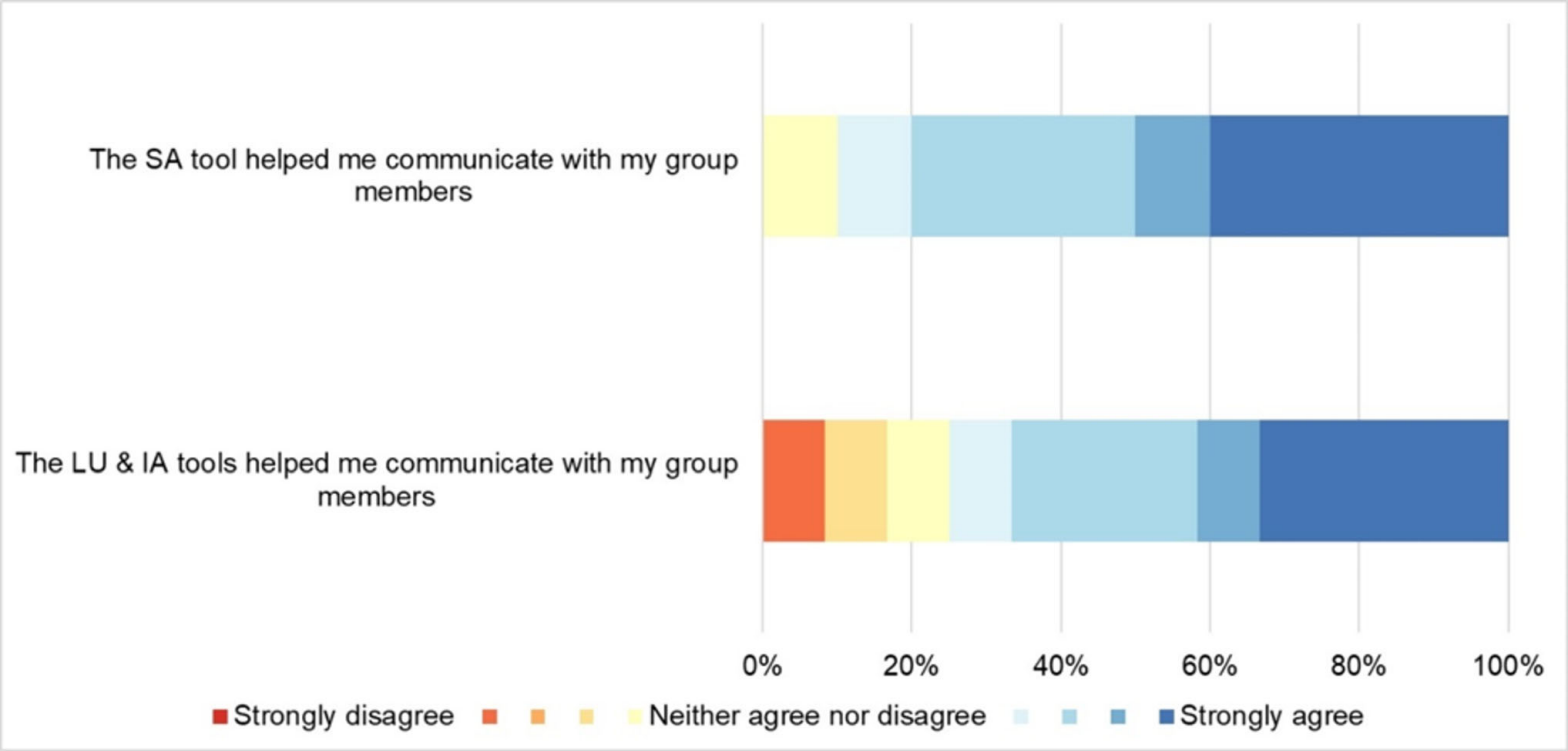
Perceived contribution of the ‘suitability analysis’ (SA) and ‘land use change and impact assessment’ (LU & IA) steps to facilitate knowledge co-production through communication

**Fig. 7:**
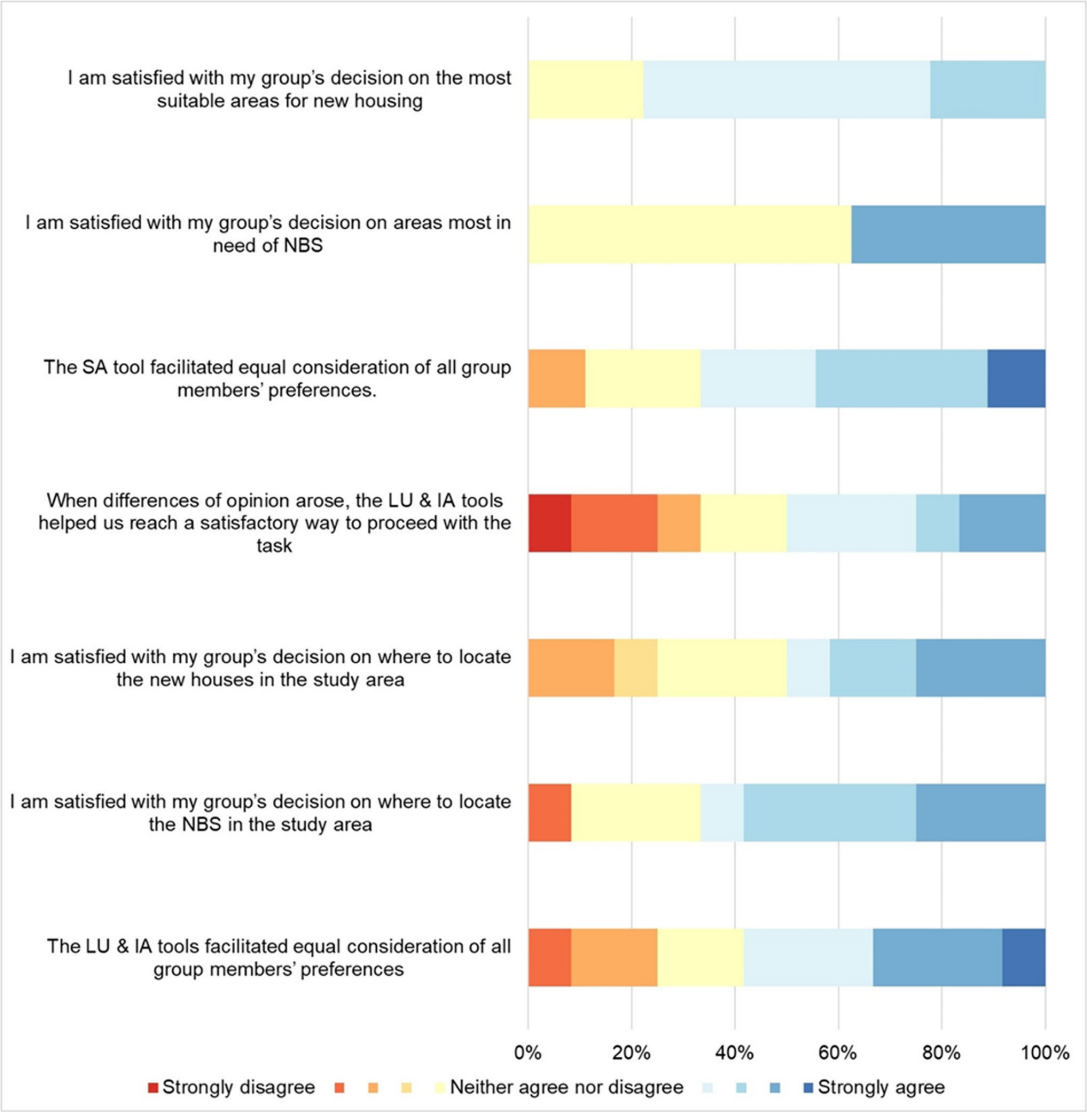
Perceived contribution of the ‘suitability analysis’ (SA) and ‘land use change and impact assessment’ (LU & IA) steps to facilitate knowledge co-production through mediation

The perceived contribution of the ‘suitability analysis’ and ‘land use change and impact assessment’ steps to facilitate knowledge co-production through *translation*, *communication*, and *mediation* is described in the following subsections and shown in Figs. 5, 6, and 7, respectively. These results are subsequently complemented with selected quotes from geodesign participants in Table 3.

**Fig. 8:**
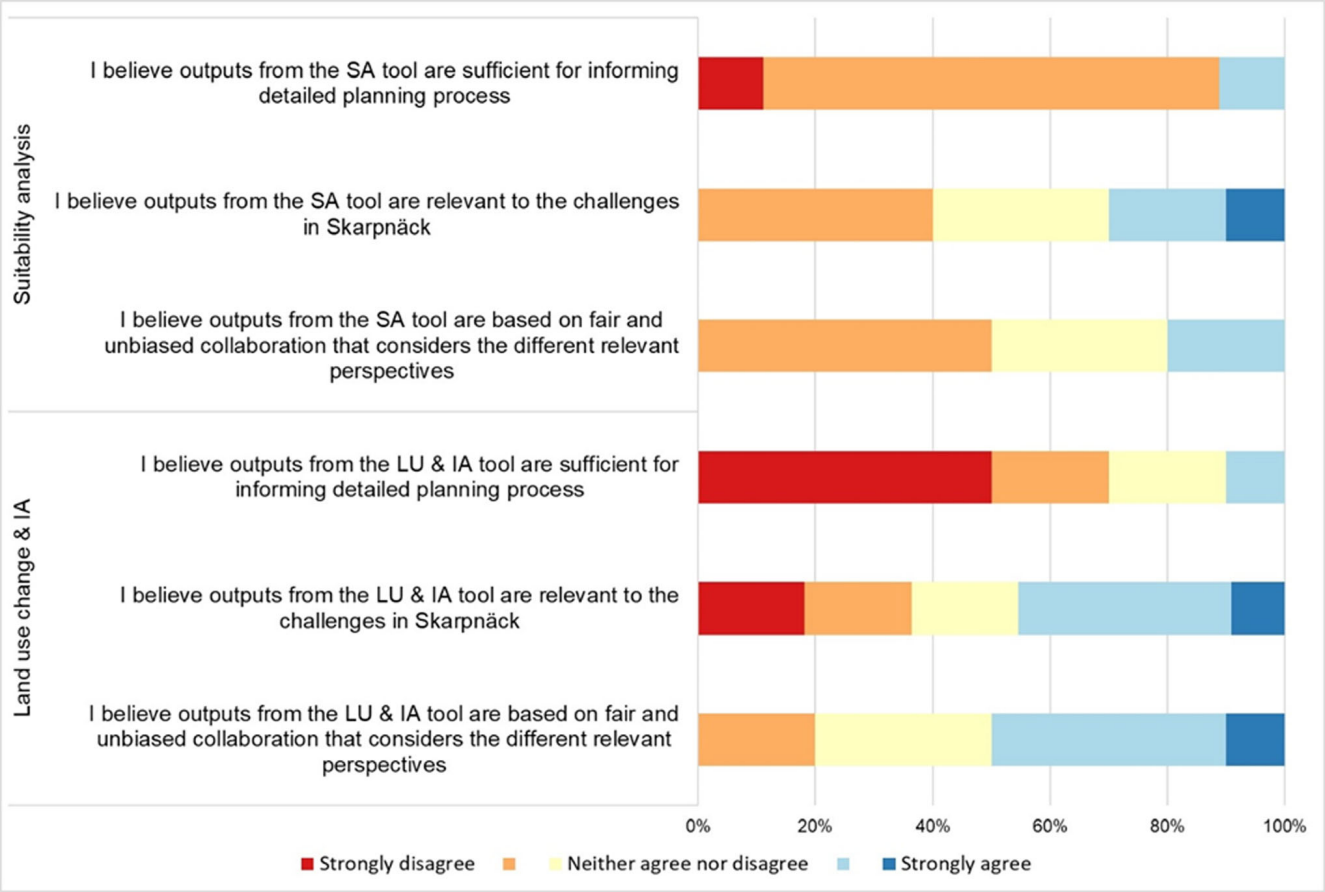
Perceived credibility, saliency, and legitimacy of the geodesign outputs as boundary objects: Suitability analysis - SA (top 3) and Land use change and Impact assessment—LU & IA (bottom 3).

**Table 3:** Selected quotes and summarized sentiments on the perceived contribution of the different geodesign steps to facilitate knowledge co-production through TCM

Suitability analysis	Land use change and impact assessment
A2 expressed concern about already working with enough tools and proposed additional criteria and adaptations for different target groups to enhance the tool’s effectiveness in bridging communication gaps	Group A participants questioned both the land-use classification and expert-based values for impact assessment (ES stormwater retention) used in the tool
A3 suggested considering up to 40 criteria, but also acknowledged practical difficulties	B1 “*It can get tricky if values are different or if paradigms are different (..). But as long as you're within the same value space, I think it works… to some extent… to facilitate the discussion and make people talk and contribute”*
A4 *“…the start of this discussion and to meet other people and to look at the same map at the same time, that kind of moment [is] not so common in the planning process*”	B4 “*In the area that we looked at… we have a lot of information already. I think all of us that work in the area know every parcel of land essentially… [but]it's overlaid some and it's good for discussion*”
B1 “*…most of all, I think it's facilitated discussion. Even if there are different views, you can more easily… listen to other people's views and maybe come to a new understanding as well*.”	C1 “*we need the whole picture! The opportunity spaces are taken out of their perspective without the different biotopes around)*.”
B2 “*I think it was good for triggering a discussion. It's good for reasoning (together)*”	C2 "*The tool is there, but you need to also consider the group dynamics so that everyone gets a chance to speak, and you cannot control the conversation with the tool*”
B4 “*I'm not entirely sure that they're more effective than a workshop without the tools with actual maps given that you need so much local knowledge to interpret the layers and the statistics to do the rating and the assumptions*.”	C3: “*Well, I will say one obvious benefit is the aim or the possibility to bring the transformative approach forcing us to think sort of out of the box, our own, our different ordinary boxes. So, to stretch out what is common detailed planning or structural planning, I think that's great!*”
B1 and C1 expressed concerns that the proposed tools did not adequately address important issues such as climate change adaptation, wetlands, and noise	
C3. “*It was hard to say which is most important: hundred meters to go to a school or hundred meters to an oak. Actually, evaluating the (criteria) was hard. The idea of using a tool is good''*	

#### Translation

Suitability Analysis (Step 3). Participants' opinions on the *translation* function (i.e., ability to combine diverse data effectively) of the suitability analysis tool for housing and NBS were mixed, with most responses clustered around the midpoint. Six responses raised ‘strong disagreement’ (2), with three referring to new housing and three to the need for NBS (i.e., A2; C1; C3; C4). Concerns focused on converting value judgments into the 1–10 scale, proposing additional criteria, and adaptations for different target groups. Some participants suggested considering up to 40 criteria, albeit acknowledging practical difficulties. Group C, which experienced difficulties with the tool, gave low evaluations (mean 2.00; 2.33) for the *translation* items, while Group B gave the highest ratings (5.67; 6.0).

##### Land use change and impact assessment (Steps 4 and 5)

The *translation* function, assessed by the tool’s ability to combine different data for land use change and impact assessment, was perceived as insufficient by most participants, with 11 strong disagreements. The tool was rated slightly better for identifying areas for NBS compared to new housing (4.42 vs 3.92). The *translation* function received the lowest scores from Group A participants (3.00; 3.00), followed by Group C (4.25; 4.75), while Group B participants gave the highest scores (4.50; 5.50). Group A participants questioned land-use classification and expert-based values for impact assessment, feeling restricted by the tool's focus on ‘opportunity spaces’ and the coarse resolution. Group B found the tool useful for discussion but noted it did not provide significantly new information.

#### Communication

##### Suitability analysis (Step 3)

Most participants found the suitability analysis tool highly effective for *communication* within their groups (mean 7.6). Only one participant (C3) had a neutral stance. Five participants (A1; B1; B2; C1; C4) strongly agreed that the tool helped them communicate with their group members, suggesting that the communication aspect of the tool was generally well-received. Participants highlighted that the tool facilitated discussions, reasoning together, and triggered new understandings (B1, B2, B4). However, one respondent (B4) expressed doubt about the added value of the tool in planning contexts, arguing that local expertise is still necessary to interpret the data.

##### Land use change and impact assessment (Steps 4 and 5)

Feedback regarding the *communication* function of the tool was mixed. On average, the tool received a relatively high rating of 6.8, indicating that it facilitated communication for most participants. However, there was no consensus, as some respondents scored it below the midpoint, and A1 remained neutral. Nonetheless, several participants (B3; C1; C2; C3; C4) strongly agreed (8–9) that the tool facilitated *communication*, promoting fruitful discussions and new perspectives. They appreciated the tool's ability to compare scenarios, offer visualization options, and stimulate innovative thinking. However, some participants disagreed, highlighting the tool's dependence on contextual factors such as group dynamics and participants' values or worldviews. This diversity in perceptions underscores the tool's potential for fostering effective *communication*, but also its sensitivity to contextual factors.

#### Mediation

##### Suitability analysis (Step 3)

The pre-task baseline indicates that the average perceived level of agreement among group members before the task was slightly below the midpoint. The potential for improved agreement was slightly higher for housing compared to the need for NBS (mean 4.20 vs. 4.90). In line with this, the post-task results suggest moderate to high *mediation* functions, with participants generally satisfied with the group decision-making process (mean 6.00 for new housing and 6.13 for the need of NBS). Only one respondent disagreed that the tool facilitated equal consideration of all group members' preferences’ (B4; ‘3’ response). Group A perceived the highest potential for mediation with a score of 6.75, while Group C perceived the lowest potential with a score of 5.

##### Land use change and impact assessment (Steps 4 and 5)

The pre-task baseline indicates that most participants perceived a high level of agreement within their groups. However, there were notable outliers (B1, B2, C1) who expressed strong disagreement, likely reflecting their dissenting views along with other external factors. The post-task survey results reveal a mixed perception of the *mediation* function, with an average score around the midpoint (4.92). Four participants (A1; A2; B1; C1) disagreed (with responses of 2 or 3) that the tool helped to reach a satisfactory resolution when differences of opinion arose during the task. Additionally, some respondents (A1; B1; C1) did not agree that the tool facilitated equal consideration of all group members' preferences, despite an average score of around 6. Based on comments from other points in the workshop, these challenges in using the tool to mediate disagreements may be attributed to the significance of group dynamics and moderation, which are beyond the tool's capacity to regulate individual contributions.

### Perceived credibility, salience, and legitimacy of the geodesign outputs

#### Credibility

##### Suitability analysis (Step 3)

The participants' showed high skepticism about the *credibility* of the suitability analysis during the session (Fig. 8). Seven out of nine participants expressed disagreement (scoring less than 3 on the 5-point Mentimeter scale) with the tool's results, indicating a lack of confidence in its suitability for detailed planning processes. Concerns were raised regarding the selection of criteria, with an emphasis on aligning them with legal requirements (A2) and political priorities (A3, A4). Participants found it challenging to weigh such diverse criteria (e.g., A3, C3), and some questioned the sensibility of the approach (A2). For example, participant C3 voiced doubts about the suitability of the suggested locations generated by the tool.

##### Land use change and impact assessment (Steps 4 and 5)

The majority of the respondents (7 out of 10) disagreed with the statement that the outputs of these two steps were sufficient for informing a typical detailed planning process (Fig. 8). Five participants had serious reservations about the *credibility* of the results, while two considered them rather moderately credible. Only one participant perceived the results as slightly credible. Participant A3 raised concerns about the expert-based evaluation of the impact assessment, specifically with regards to the weighting of different biotopes based on their ES potential. Additionally, A3 highlighted how the tool may obscure uncertainty, as its precision depends on the input of 'expert values', which may not be precise. C3 summarized the challenge of dealing with multiple models that cover different aspects and suggested that combining various parameters and inputs from specialized programs could be a more effective approach, emphasizing the limitations of a single tool in performing all calculations.

#### Saliency

##### Suitability analysis (Step 3)

Participants held varying opinions on how relevant the outputs of the suitability analysis tool were to address the societal challenges in the study area (Fig. 8). Four out of ten disagreed, three agreed, and three remained neutral. For instance, B1 noted that while some aspects of the results were relevant, the tool did not fully address critical aspects such as climate change adaptation. A2 highlighted the influence of criteria selection and interpretation on output relevance, suggesting a need for tailoring criteria to specific planning questions. In fact, A2 and B4 emphasized the tool's potential to demonstrate the ‘complexity’ of planning to politicians and the public, and to serve as a bridge between different stakeholders, including developers.

##### Land use change and impact assessment (Steps 4 and 5)

The participants had mixed but slightly agreeing perceptions regarding the relevance of geodesign outputs to the study area’s challenges (Fig. 8). While one participant strongly agreed, two participants strongly disagreed with the relevance of the land use and impact assessment outputs. Several participants noted a key limitation of the tool, which is its inflexibility in addressing the potential of the entire area due to inability to make changes beyond predefined ‘opportunity spaces’ (e.g., A2, A3, B2, C1). Of note, C2 proposed using the tool for smaller-scale detailed plans, rather than covering an entire neighborhood, and highlighted its value in making comparisons with the current situation (C2). C3 emphasized the tool's role in highlighting issues in the study area but not providing definitive solutions, underlining its importance as a support mechanism rather than a final planning solution.

#### Legitimacy

##### Suitability analysis (Step 3)

The legitimacy of the outputs was not strongly agreed upon by the participants (Fig. 8). Out of ten respondents, five disagreed that the outputs were based on fair and unbiased collaboration that considered different perspectives. Only two participants perceived the legitimacy of the outputs positively, while three participants remained neutral. B4 questioned the tool's role in ensuring legitimacy compared to a workshop with traditional paper maps, emphasizing the need for local knowledge to interpret the tool's layers, statistics, and assumptions. A3, on the other hand, argued that *“For the discussions, I think it was better than just to have different maps’.* In addition, A3 stressed the importance of the group composition, noting that the legitimacy of outputs depends on having a diverse group that reflects the perspectives of politicians and stakeholders.

#### Land use change and impact assessment (Steps 4 and 5)

The overall perception of the legitimacy of the geodesign outputs was positive (Fig. 8). Five out of eleven respondents agreed that the outputs were the result of a fair and unbiased collaboration that considered different perspectives. Only two participants had minor doubts, and three participants expressed indifference. C2 highlighted again the role of group dynamics in determining the legitimacy of the outputs, noting that the tool alone cannot steer the process. B1 acknowledged that the tool can facilitate discussions and collaboration but pointed out that challenges may arise when values and paradigms differ among participants. Despite the challenges, B1 observed that the tool can be effective when participants share similar values and worldviews.

## Discussion

### Effectiveness of geodesign for boundary work

Our study investigated participants’ perceptions throughout the whole geodesign process, providing a nuanced understanding of the different steps and their boundary work functions. Boundary work is key to facilitating stakeholder collaboration for effectively integrating, interpreting, and communicating planning process input data and outcomes. As shown by Gottwald et al., geodesign can help manage "boundaries" as shared dynamic interfaces where stakeholders collaboratively address planning and management challenges (Gottwald et al. 2021a, b). Their study argues that geodesign effectively supports boundary work's *translation*, *communication*, and *mediation* functions. Similarly, Schröter et al., (2023a) indicated that the geodesign tool was valuable and supportive to local stakeholders. Building on these insights, our study revealed the strengths and limitations of the different geodesign steps in terms of both TCM and CSL.

The analysis of participants' responses revealed varying scores across TCM functions, with *communication* generally receiving higher scores, while *translation* and *mediation* functions scored lower and showed more variability across different steps of the process. These differences in scoring provide valuable insights into the tool's effectiveness in different aspects of the planning process.

Participants held varied opinions on the tool's *translation* function, intended as the ability to combine diverse data effectively. Particularly, for the suitability analysis (Step 3), concerns arose regarding criteria selection and weighting, as well as the tool's suggested locations for new housing and NBS, prompting discussions on input data and the need for comprehensive information before assigning weights. In the land use change and impact assessment (Steps 4 and 5), most participants found the tool's *translation* function inadequate.

Despite these *translation* challenges, the tool's *communication* aspect showed more promise. Participants generally found the tool effective for facilitating group discussions, especially during the suitability analysis step. This dichotomy between *translation* and *communication* effectiveness suggests that even when a tool struggles to fully capture complex data, it can still be a valuable platform for fostering dialogue and shared understanding among stakeholders.

The *mediation* function showed mixed results across different steps, reflecting the complex dynamics of group decision-making processes. This variability in *mediation* effectiveness shows the tool's sensitivity to contextual factors such as group dynamics and individual perspectives, underscoring the importance of considering these elements in geodesign processes. Among others, this highlights the potential of geodesign analytics to acquire a nuanced comprehension of group dynamics throughout the design process (Freitas and Moura 2018; Cocco et al. 2019b, 2019a). When displayed in real time through dashboards, log-data gathered by web-based collaborative platforms can facilitate, for instance, the addressing of power imbalances by facilitators and encourage participants to become more reflective of each other's roles.

Regarding the boundary work criteria of CSL, our study revealed several critical insights. Participants expressed skepticism about the credibility of both the suitability analysis and the land use change and impact assessment outputs. This skepticism stemmed from concerns about criteria selection, data quality, and the challenge of balancing diverse factors in complex planning decisions. For example, stakeholders observed that around 40–50 factors derived from existing plans, regulations, policies, and standards would need to be incorporated into the GIS interface to accurately represent the assessment carried out within a real-life planning process. In the following steps, the expectations of planners directly involved in ongoing detailed planning processes did not align with simplified impact assessments allowed by the geodesign application, and participants suggested integrating parameters and inputs from specialized programs to adhere more closely to their knowledge of reality. These findings highlight the critical role of data quality and processing in perceived tool credibility, especially for knowledgeable participants. Transparency was also identified as a key aspect that improves the legitimacy and credibility of the geodesign process. For example, in land use change and impact assessment (Steps 4 and 5), most respondents doubted the results' adequacy for informing detailed planning, citing concerns about credibility and potential uncertainty masking and emphasizing the importance of acknowledging this uncertainty in decision-making rather than oversimplifying complex assessments.

While valuable in providing stakeholders with data-driven insights, our study confirmed earlier findings (e.g., Gottwald et al. 2021a) on the common challenge of aggregating information in geodesign tools and data simplification for faster calculation. Striking the right balance between the level of detail and speed of analysis is a critical challenge in geodesign. Sacrificing detail may be acceptable for tasks like suitability analysis, where strategic decisions on what factors to include, if agreed upon by the participants, can even help to maintain the legitimacy and credibility of results, which tend to diminish when more complexity is introduced into the model (Cortinovis and Geneletti 2020; Gottwald et al. 2021a, b). In our case, we observed that despite potential concerns about accuracy, the tool facilitated the sharing of perspectives and knowledge among participants from different planning levels, creating a shared 'map' of challenges and opportunities for new housing and NbS as a form of ‘boundary object’. On the other hand, excessive simplification can hinder capturing nuances in land use change impacts. Our study prioritized tool performance to support real-time discussions in face-to-face digital workshops where different alternatives must be formulated and compared. However, the possibility to act only at the level of the mapping units of the biotope data (Skånes 2022) was perceived by participants as suboptimal in many cases. Overall, the findings underscore the need to tailor simplification levels to each geodesign process's specific needs, striking the right balance between complexity and feasibility, which is particularly challenging when operating within tight timeframes (Gottwald et al. 2021a, b; Campagna 2022).

In terms of salience, the tool's outputs also received mixed reviews, with participants holding varying opinions on its effectiveness in addressing the study area's challenges. Despite this, with regard to suitability analysis (Step 3), participants recognized the tool's potential to communicate planning complexity to stakeholders like politicians and developers. Similarly, in the land use change and impact assessment (Steps 4 and 5), opinions on the relevance of geodesign outputs varied, with some strongly disagreeing and others strongly agreeing. A key limitation reported was the tool's inflexibility and focus on predefined "opportunity spaces". Some participants suggested using it for smaller-scale plans and emphasized its role in highlighting rather than providing definitive solutions. Indeed, participants valued the outputs but stressed the need for flexibility, interactivity, and scalability to address complex challenges effectively.

In terms of legitimacy, perceptions were also varied, with more positive views for the land use change and impact assessment steps compared to the suitability analysis. Group dynamics were seen as crucial, with the understanding that the tool alone cannot guarantee legitimacy. As such, challenges may arise from differing values and worldviews, but the tool can help to build shared perspectives. For example, officials from the Stockholm County Administrative Board, tasked with formulating the county-level green infrastructure action plan, discussed with the municipal planners how to interpret relevant information, such as habitat areas (without protection) and their assumed connectivity zones. This could mitigate potential discrepancies in expectations among planners at adjacent levels, as highlighted by Högström et al., (2018), thereby potentially fostering synergy among planning instruments.

These findings align with and extend previous studies, such as the Lahn River case study (Gottwald et al. 2021b, 2021a), where participants expressed mixed opinions on geodesign effectiveness. Our study provides a more detailed understanding of the strengths and limitations across different geodesign steps, offering insights for future improvements in geodesign tools and processes. It underscores the potential of geodesign as a valuable tool for communication, visualization, and plan evaluation, while also highlighting areas for improvement in data integration, flexibility, and stakeholder engagement.

Finally, our selection of the tool (and related data) played a significant role in influencing the participants' responses. The selection of CommunityViz was based on both our own and others’ previous experience with the tool: a number of international case studies have employed CommunityViz in geodesign processes at varying scales, thereby demonstrating its extensive applicability in diverse contexts (Janssen et al. 2014; Pettit et al. 2019; Gottwald et al. 2021b). Indeed, assessing boundary work effectiveness in geodesign applications using different digital tools or platforms would provide useful information on their suitability to different contexts and decision-making issues.

In terms of content, our study confirms that urban planning with NBS necessitates the integration of diverse data and knowledge, as well as the facilitation of transdisciplinary collaboration through boundary work. This makes geodesign a valuable methodological approach for advancing boundary work for urban planning with NBS to address *wicked problems*. It is acknowledged that the integration of housing and other gray infrastructures with green spaces in planning with geodesign has been explored in existing literature (e.g., Campagna et al. 2020; Rolf and Peters 2020). Our study underscores the necessity for further empirical applications to fully harness the potential of geodesign in addressing complex challenges such as providing new housing while addressing issues of biodiversity loss, climate change, and urban quality of life. By implementing geodesign in tangible, real-world scenarios, we can evaluate the associated boundary work to gain a deeper understanding of its capabilities and limitations, thereby advancing the field and enhancing its utility for urban planners and stakeholders.

### Geodesign metaplanning integrating boundary work assessment

Integrating the structure of the geodesign process with a protocol for assessing boundary work functions and criteria is one of the main innovative aspects of the present study. The integrated conceptual framework presented in Sect. "Geodesign process and boundary work: A conceptual framework" and its operationalization through the methods for eliciting participants’ feedback described in Sect. "Conducting the workshop and collecting participants’ feedback" are a first attempt to systematically incorporate the assessment of boundary work as a replicable protocol for geodesign applications. The basis of the framework consists of a scenario-based geodesign process composed of six core steps, which build on previous research in the field of NBS (Albert et al. 2021; Gottwald et al. 2021b). Each step of the process produces an output that can be considered a *boundary object*, hence assessed by the participants in terms of *credibility*, *salience*, and *legitimacy* (Cash et al. 2003; Mitchell et al. 2006). Moreover, interactions throughout the process should promote the boundary functions of *translation*, *communication*, and *mediation* (Cash et al. 2003; Clark et al. 2016). Evaluating the extent to which each step contributes to the three functions is crucial for understanding the effectiveness of the geodesign exercise in bridging different perspectives.

The proposed conceptual framework responds to the call for geodesign metaplanning launched by Campagna, (2016). The author highlighted the importance and benefits of “designing the geodesign process” to expand the tasks already described in the second iteration of Steinitz’s geodesign framework (Steinitz 2012). According to Campagna, metaplanning entails not only selecting the most appropriate methods for the geodesign application, but also defining the role of each actor, their activities, and the expected products and results by (Campagna 2016). The proposed framework further expands these tasks by explicitly including the assessment of boundary work as a key component of the geodesign process. The importance of planning processes evaluation has already been stressed (Guyadeen and Seasons 2018), especially in relation to participation (Laurian and Shaw 2009). Previous assessments of geodesign processes demonstrated the usefulness of assessing indicators associated with the perception of participants not only to identify areas of improvement but also as a way to enhance the participatory experience of the whole process (Kuniholm 2020). Therefore, identifying key aspects to investigate and suitable methods to evaluate the effectiveness of boundary work within the process should be considered as an additional task of geodesign metaplanning.

From the methodological point of view, previous research has employed ad hoc surveys and short discussion prompts closely aligned with the specific contextual goals of the individual projects involving boundary work that were analyzed (Cutts et al. 2011; Kuniholm 2020; André et al. 2021). Our questionnaires and FGD prompts were designed to cover different dimensions of the broader concepts identified in past research, using several key papers (e.g., Cash et al. 2003) as a basis. Items and prompts were created with core statements and context-specific terms added to effectively convey meanings to stakeholders. Future research could adapt the items and prompts to other contexts and test them further with larger samples. In this way, it would be possible to explore and ultimately improve the validity of (aggregated) items related to dimensions of the same concept. Using validity-tested scales facilitates greater standardization in the field of boundary work research, enabling comparison between cases and over time.

In our research, triangulating between comments, survey responses, and transcripts suggests that items and prompts mostly performed well at eliciting perceptions of TCM and CSL. However, several comments also point at aspects in need of further improvement. For instance, regarding the assessment of the potential for *mediation* as a baseline measure, one respondent (A1) indicated that they were previously unaware of the extent of consensus regarding the most suitable areas within their group before using the aforementioned tools. Determining baseline values through alternative methodologies or creating a proxy score based on a more comprehensive understanding of the individuals, their backgrounds, etc., could prove to be a valuable approach.

Despite these limitations, integrating the protocol to assess stakeholders’ perception of TCM and CSL into the geodesign process provided useful insights on several aspects of the process and of its implementation, including but not limited to the technical aspects. Further refinements and testing could finetune the surveys and discussion prompt and explore synergies and potential integrations with alternative ways of capturing geodesign dynamics. For example, log-data gathered by web-based collaborative platforms allow a quantitative analysis of workshop dynamics (Cocco et al. 2019b; Cocco and Campagna 2020), which could complement the qualitative information gathered through surveys and FGD.

## Conclusions

Our findings indicate that the geodesign process worked well in enhancing *communication* among participants and served as a valuable tool for initiating discussions and collective reasoning at each step. Participants acknowledged the role of the geodesign process in aiding knowledge co-production and decision-making by *mediating* between different perspectives and opinions, with the suitability analysis step having a slightly stronger impact in this regard. However, some reservations were expressed regarding the *translation* aspect of knowledge co-production. Concerning this, participants indicated skepticism toward the suitability analysis, land use change, and impact assessment tools.

In conclusion, three key lessons emerge. First, co-defining the setting with local partners helps ensure that the most relevant issues are addressed and that models fit their understanding, thereby promoting transparency. This key step can be associated with the first and second iterations of the Steinitz Geodesign framework that refer to "Understand the study area" and "Specify method". Second, there is need to improve geodesign and skills through better tools, training, and experiences. While this may seem straightforward, it reflects a critical gap in the mainstream application of geodesign in Swedish context and beyond. Despite the emerging literature on geodesign applications, geodesign remains a niche within real-world planning practices. Mainstreaming geodesign will require tools that are more user-friendly and tailored to the practical needs of planners and stakeholders, as well as comprehensive training programs. Our case study highlights these needs and underscores the importance of integrating advanced tools and training to enhance the effectiveness of geodesign in urban planning. Third, geodesign applications should capitalize on the potential to foster integrated urban planning, simultaneously considering settlement and NBS development, thereby minimizing conflicts and exploiting synergies through well-informed plan- and decision-making. It is our hope that the method presented in this paper, and the insights generated from its application, will prove a valuable resource for those seeking to develop future geodesign applications in other contexts.

## Supplementary Information

Below is the link to the electronic supplementary material.

Supplementary file1 (PDF 3033 KB)

## References

[CR300] Adams, C., N. Frantzeskaki, and M. Moglia. 2023. Mainstreaming nature-based solutions in cities: A systematic literature review and a proposal for facilitating urban transitions. *Land Use Policy* 130: 106661. 10.1016/j.landusepol.2023.106661.

[CR1] Adem Esmail, B., and D. Geneletti. 2017. Design and impact assessment of watershed investments: An approach based on ecosystem services and boundary work. *Environmental Impact Assessment Review* 62: 1–13. 10.1016/j.eiar.2016.08.001.

[CR2] Adem Esmail, B., D. Geneletti, and C. Albert. 2017. Boundary work for implementing adaptive management: A water sector application. *Science of the Total Environment* 593–594: 274–285. 10.1016/j.scitotenv.2017.03.121. 10.1016/j.scitotenv.2017.03.121 28346901

[CR3] Adem Esmail, B., C. Cortinovis, L. Suleiman, C. Albert, D. Geneletti, and U. Mörtberg. 2022. Greening cities through urban planning: A literature review on the uptake of concepts and methods in Stockholm. *Urban Forestry & Urban Greening* 72: 127584. 10.1016/j.ufug.2022.127584.

[CR4] Albert, C., J. Aronson, C. Fürst, and P. Opdam. 2014. Integrating ecosystem services in landscape planning: Requirements, approaches, and impacts. *Landscape Ecology* 29: 1277–1285. 10.1007/s10980-014-0085-0.

[CR5] Albert, C., M. Brillinger, P. Guerrero, S. Gottwald, J. Henze, S. Schmidt, E. Ott, and B. Schröter. 2021. Planning nature-based solutions: Principles, steps, and insights. *Ambio* 50: 1446–1461. 10.1007/s13280-020-01365-1. 33058009 10.1007/s13280-020-01365-1 PMC8249551

[CR6] Anderson, C.C., and F.G. Renaud. 2021. A review of public acceptance of nature-based solutions: The ‘why’, ‘when’, and ‘how’ of success for disaster risk reduction measures. *Ambio* 50: 1552–1573. 10.1007/s13280-021-01502-4. 33606249 10.1007/s13280-021-01502-4 PMC8249538

[CR7] André, K., L. Järnberg, Å. Gerger Swartling, P. Berg, D. Segersson, J.H. Amorim, and L. Strömbäck. 2021. Assessing the quality of knowledge for adaptation-experiences from co-designing climate services in Sweden. *Frontiers in Climate* 3: 1–12. 10.3389/fclim.2021.636069.

[CR8] Babí Almenar, J., T. Elliot, B. Rugani, B. Philippe, T. Navarrete Gutierrez, G. Sonnemann, and D. Geneletti. 2021. Nexus between nature-based solutions, ecosystem services and urban challenges. *Land Use Policy* 100: 104898. 10.1016/j.landusepol.2020.104898.

[CR9] Bartel, C.A., and R. Garud. 2009. The role of narratives in sustaining organizational innovation. *Organization Science* 20: 107–117. 10.1287/orsc.1080.0372.

[CR10] Brokking, P., U. Mörtberg, and B. Balfors. 2021. Municipal practices for integrated planning of nature-based solutions in urban development in the stockholm region. *Sustainability* 13: 1–20. 10.3390/su131810389.

[CR11] Brown, V.A., J.A. Harris, and J.Y. Russell, eds. 2010. *Tackling wicked problems through the transdisciplinary imagination*. London: Earthscan.

[CR12] CAB. 2019. *Stockholm County Administrative Board. Grön Infrastruktur. Regional Handlingsplan för Stockholms Län (Fastställd November 2018)*. Stockholm, Sweden: Stockholm County Administrative Board.

[CR13] Campagna, M. 2016. Metaplanning: About designing the Geodesign process. *Landscape and Urban Planning* 156: 118–128. 10.1016/j.landurbplan.2015.08.019.

[CR14] Campagna, M. 2022. Geodesign in the planning practice: Lessons learnt from experience in Italy. *Journal of Digital Landscape Architecture* 2022: 496–503. 10.14627/537724048.

[CR15] Campagna, M., and E.A. Di Cesare. 2016. Geodesign: Lost in Regulations (and in Practice). *Green Energy and Technology* 0: 307–327. 10.1007/978-3-319-31157-9_16.

[CR16] Campagna, M., E.A. Di Cesare, and C. Cocco. 2020. Integrating green-infrastructures design in strategic spatial planning with geodesign. *Sustainability* 12: 1820. 10.3390/su12051820.

[CR17] Cash, D.W., W.C. Clark, F. Alcock, N.M. Dickson, N. Eckley, D.H. Guston, J. Jager, and R.B. Mitchell. 2003. Knowledge systems for sustainable development. *Proceedings of the National Academy of Sciences* 100: 8086–8091. 10.1073/pnas.1231332100. 10.1073/pnas.1231332100 PMC166186 12777623

[CR18] Clark, W.C., T.P. Tomich, M. van Noordwijk, D. Guston, D. Catacutan, N.M. Dickson, and E. McNie. 2016. Boundary work for sustainable development: Natural resource management at the Consultative Group on International Agricultural Research (CGIAR). *Proceedings of the National Academy of Sciences* 113: 4615–4622. 10.1073/pnas.0900231108. 10.1073/pnas.0900231108 PMC4855572 21844351

[CR19] Cocco, C., and M. Campagna. 2020. A quantitative approach to geodesign process analysis. *Journal of Digital Landscape Architecture* 2020: 432–438. 10.14627/537690044.

[CR20] Cocco, C., P. Jankowski, and M. Campagna. 2019a. An analytic approach to understanding process dynamics in geodesign studies. *Sustainability* 11: 4999. 10.3390/su11184999.

[CR21] Cocco, C., C. Rezende Freitas, A.C. Mourão Moura, and M. Campagna. 2019b. Geodesign process analytics: Focus on design as a process and its outcomes. *Sustainability* 12: 119. 10.3390/su12010119.

[CR22] Collier, M.J., Z. Nedović-Budić, J. Aerts, S. Connop, D. Foley, K. Foley, D. Newport, S. McQuaid, et al. 2013. Transitioning to resilience and sustainability in urban communities. *Cities*. 10.1016/j.cities.2013.03.010.

[CR23] Cortinovis, C., and D. Geneletti. 2020. A performance-based planning approach integrating supply and demand of urban ecosystem services. *Landscape and Urban Planning* 201: 103842. 10.1016/j.landurbplan.2020.103842.

[CR24] Crona, B.I., and J.N. Parker. 2012. Learning in support of governance: Theories, methods, and a framework to assess how bridging organizations contribute to adaptive resource governance. *Ecology and Society* 17: 32. 10.5751/ES-04534-170132.

[CR25] Cutts, B.B., D.D. White, and A.P. Kinzig. 2011. Participatory geographic information systems for the co-production of science and policy in an emerging boundary organization. *Environmental Science and Policy* 14: 977–985. 10.1016/j.envsci.2011.05.012.

[CR26] Dabović, T. 2020. Geodesign meets its institutional design in the cybernetic loop. *Journal of Digital Landscape Architecture* 2020: 486–496. 10.14627/537690050.

[CR27] EU Copernicus LMS, and EEA. 2018. Copernicus impreviosness data. EU Copernicus Land Monitoring Service European Environment Agency.

[CR28] Frantzeskaki, N. 2019. Seven lessons for planning nature-based solutions in cities. *Environmental Science and Policy* 93: 101–111. 10.1016/j.envsci.2018.12.033.

[CR29] Frantzeskaki, N., P. Vandergert, S. Connop, K. Schipper, I. Zwierzchowska, M. Collier, and M. Lodder. 2020. Examining the policy needs for implementing nature-based solutions in cities: Findings from city-wide transdisciplinary experiences in Glasgow (UK), Genk (Belgium) and Poznań (Poland). *Land Use Policy* 96: 104688. 10.1016/j.landusepol.2020.104688.

[CR30] Freitas, C.R., and A.C.M. Moura. 2018. ETL tools to analyze diagrams’ performance: Favoring negotiations in geodesign workshops. *Disegnarecon.* 11: 15.1-15.22.

[CR31] Geels, F.W. 2011. The multi-level perspective on sustainability transitions: Responses to seven criticisms. *Environmental Innovation and Societal Transitions* 1: 24–40. 10.1016/j.eist.2011.02.002.

[CR32] Geertman, S., and J. Stillwell. 2004. Planning support systems: An inventory of current practice. *Computers, Environment and Urban Systems* 28: 291–310. 10.1016/S0198-9715(03)00024-3.

[CR33] Geertman, S., and J. Stillwell. 2020. Planning support science: Developments and challenges. *Environment and Planning b: Urban Analytics and City Science* 47: 1326–1342. 10.1177/2399808320936277.

[CR34] Geneletti, D., C. Cortinovis, L. Zardo, and B. Adem Esmail. 2020. Planning for ecosystem services in cities. *SpringerBriefs in Environmental Science. Cham: Springer International Publishing.*10.1007/978-3-030-20024-4.

[CR35] Gottwald, S., J. Brenner, C. Albert, and R. Janssen. 2021a. Integrating sense of place into participatory landscape planning: merging mapping surveys and geodesign workshops. *Landscape Research* 46: 1041–1056. 10.1080/01426397.2021.1939288.

[CR36] Gottwald, S., J. Brenner, R. Janssen, and C. Albert. 2021b. Using Geodesign as a boundary management process for planning nature-based solutions in river landscapes. *Ambio* 50: 1477–1496. 10.1007/s13280-020-01435-4. 33331977 10.1007/s13280-020-01435-4 PMC8249630

[CR37] Guyadeen, D., and M. Seasons. 2018. Evaluation theory and practice: Comparing program evaluation and evaluation in planning. *Journal of Planning Education and Research* 38: 98–110. 10.1177/0739456X16675930.

[CR38] Högström, J., B. Balfors, and M. Hammer. 2018. Planning for sustainability in expansive metropolitan regions: Exploring practices and planners’ expectations in Stockholm, Sweden. *European Planning Studies* 26: 439–457. 10.1080/09654313.2017.1391751.

[CR39] Hooper, P., C. Boulange, G. Arciniegas, S. Foster, J. Bolleter, and C. Pettit. 2021. Exploring the potential for planning support systems to bridge the research-translation gap between public health and urban planning. *International Journal of Health Geographics* 20: 1–17. 10.1186/s12942-021-00291-z. 34407828 10.1186/s12942-021-00291-z PMC8371821

[CR40] Huang, G., and N. Zhou. 2016. Geodesign in Developing Countries: The example of the Master Plan for Wulingyuan National Scenic Area, China. *Landscape and Urban Planning* 156: 81–91. 10.1016/j.landurbplan.2016.05.014.

[CR41] Iwaniec, D.M., E.M. Cook, M.J. Davidson, M. Berbés-Blázquez, M. Georgescu, E.S. Krayenhoff, A. Middel, D.A. Sampson, et al. 2020. The co-production of sustainable future scenarios. *Landscape and Urban Planning* 197: 103744. 10.1016/j.landurbplan.2020.103744.

[CR42] Janssen, R., S. Knudsen, V. Todorova, and A.G. Hoşgör. 2014. Managing Rapana in the Black Sea: Stakeholder workshops on both sides. *Ocean and Coastal Management* 87: 75–87. 10.1016/j.ocecoaman.2013.10.015.

[CR43] Kauark-Fontes, B., L. Marchetti, and F. Salbitano. 2023. Integration of nature-based solutions (NBS) in local policy and planning toward transformative change: Evidence from Barcelona, Lisbon, and Turin. *Ecology and Society*. 10.5751/ES-14182-280225.

[CR44] Kuniholm, M. 2020. Evaluating participatory and technological integration in geodesign practice. *Journal of Digital Landscape Architecture* 2020: 439–446. 10.14627/537690045.

[CR45] Lantmäteriet. 2022. GSD Property Map. Lantmäteriet.

[CR46] Laurian, L., and M.M. Shaw. 2009. Evaluation of public participation. *Journal of Planning Education and Research* 28: 293–309. 10.1177/0739456X08326532.

[CR47] Malczewski, J. 1999. *GIS and Multicriteria Decision Analysis*. New York: John Wiley & Sons.

[CR48] McDonald, R.I., A.V. Mansur, F. Ascensão, M. Colbert, K. Crossman, T. Elmqvist, A. Gonzalez, B. Güneralp, et al. 2020. Research gaps in knowledge of the impact of urban growth on biodiversity. *Nature Sustainability* 3: 16–24. 10.1038/s41893-019-0436-6.

[CR49] McPhearson, T., N. Kabisch, and N. Frantzeskaki, eds. 2023. *Nature-Based Solutions for Cities*, 1st ed. Cheltenham: Edward Elgar Publishing. 10.4337/9781800376762.

[CR50] Mitchell, R. B., W. C. Clark, D. W. Cash, and N. M. Dickson. 2006. *Global Environmental Assessments: Information And Influence*. Edited by Ronald B. Mitchell, William C. Clark, David W. Cash, and Nancy M. Dickson. Cambridge, MA: MIT Press.

[CR51] Mollinga, P.P. 2010. Boundary work and the complexity of natural resources management. *Crop Science*. 10.2135/cropsci2009.10.0570.

[CR52] Moura, A.C.M., and C.R. Freitas. 2021. Scalability in the application of geodesign in Brazil: Expanding the Use of the Brazilian geodesign platform to metropolitan regions in transformative-learning planning. *Sustainability* 13: 6508. 10.3390/su13126508.

[CR53] Mukherjee, N., J. Hugé, W.J. Sutherland, J. Mcneill, M. Van Opstal, F. Dahdouh-Guebas, and N. Koedam. 2015. The Delphi technique in ecology and biological conservation: Applications and guidelines. *Methods in Ecology and Evolution* 6: 1097–1109. 10.1111/2041-210X.12387.

[CR54] Orland, B. 2016. Geodesign to tame wicked problems. *Journal of Digital Landscape Architecture* 2016: 187–197. 10.14627/537612022.

[CR55] Parker, J., and B. Crona. 2012. On being all things to all people: Boundary organizations and the contemporary research university. *Social Studies of Science* 42: 262–289. 10.1177/0306312711435833.

[CR56] Pettit, C.J., S. Hawken, C. Ticzon, S.Z. Leao, A.E. Afrooz, S.N. Lieske, T. Canfield, H. Ballal, et al. 2019. Breaking down the silos through geodesign: Envisioning Sydney’s urban future. *Environment and Planning b: Urban Analytics and City Science* 46: 1387–1404. 10.1177/2399808318812887.

[CR57] Raymond, C.M., N. Frantzeskaki, N. Kabisch, P. Berry, M. Breil, M.R. Nita, D. Geneletti, and C. Calfapietra. 2017. A framework for assessing and implementing the co-benefits of nature-based solutions in urban areas. *Environmental Science and Policy* 77: 15–24. 10.1016/j.envsci.2017.07.008.

[CR58] Rivero, R., A. Smith, B. Orland, J. Calabria, H. Ballal, C. Steinitz, R. Perkl, L. McClenning, et al. 2017. Multiscale and multijurisdictional Geodesign: The Coastal Region of Georgia, USA. *Landscapes* 19: 42–49.

[CR59] Rolf, W., and D.G. Peters. 2020. Algorithmic landscapes meet geodesign for effective green infrastructure planning: Ideas and perspectives. *Journal of Digital Landscape Architecture* 2020: 476–485. 10.14627/537690049.

[CR60] Sarabi, S., Q. Han, B. de Vries, and A.G.L. Romme. 2022. The nature-based solutions planning support system: A playground for site and solution prioritization. *Sustainable Cities and Society* 78: 103608. 10.1016/j.scs.2021.103608.

[CR61] Schröter, B., S. Gottwald, K. Castro-Arce, E. Hartkopf, B. Aguilar-González, and C. Albert. 2023. Virtual participatory mapping of nature-based solutions in the Grande de Tárcoles River basin, Costa Rica: Connecting diverse knowledge systems in a context of physical immobility. *Science of the Total Environment.* 872: 162195. 10.1016/j.scitotenv.2023.162195. 36781131 10.1016/j.scitotenv.2023.162195

[CR62] Seto, K.C., B. Güneralp, and L.R. Hutyra. 2012. Global forecasts of urban expansion to 2030 and direct impacts on biodiversity and carbon pools. *Proceedings of the National Academy of Sciences of the United States of America* 109: 16083–16088. 10.1073/pnas.1211658109. 22988086 10.1073/pnas.1211658109 PMC3479537

[CR63] Skånes, H. 2022. Biotop SE Del B. Klassificeringssystem och databasdesign. Metadata till BIOTOP SE 4.0 (DMB220630). Stockholm: Stockholm University.

[CR64] Stockholm City. 2022. Data trädkronor [Tree canopy data]. Stockholms stad.

[CR65] Ståhle, A. 2003. Sociotopkarta för parker och andra friytor i Stockholms innerstad- om metoden, dialogen och resultatet. Stadsbyggnadskontoret och Gatu- och fastighetskontoret. Stockholm: Stockholms stad.

[CR66] Star, S.L., and J.R. Griesemer. 1989. Institutional Ecology, `translations’ and boundary objects: Amateurs and professionals in Berkeley’s museum of vertebrate zoology, 1907–39. *Social Studies of Science* 19: 387–420. 10.1177/030631289019003001.

[CR67] Steinitz, C. 2012. *A Framework for Geodesign: Changing Geography by Design*. ESRI.

[CR68] Suleiman, L., and A. Khakee. 2017. Rethinking water reform policies as a ‘wicked problem’ the case of urban water supply in Ghana. *International Planning Studies* 22: 320–332. 10.1080/13563475.2017.1291333.

[CR69] van Kerkhoff, L., and L. Lebel. 2006. Linking Knowledge and Action for Sustainable Development. *Annual Review of Environment and Resources* 31: 445–477. 10.1146/annurev.energy.31.102405.170850.

